# The role of immune cells and inflammation in pulmonary hypertension: mechanisms and implications

**DOI:** 10.3389/fimmu.2024.1374506

**Published:** 2024-03-11

**Authors:** Hui Zhao, Jialin Song, Xiujun Li, Zhaoyi Xia, Qian Wang, Jiaqi Fu, Yuqing Miao, Dapeng Wang, Xuguang Wang

**Affiliations:** ^1^ School of Materials and Chemistry, Institute of Bismuth and Rhenium, University of Shanghai for Science and Technology, Shanghai, China; ^2^ Department of Limb Trauma, Wendeng Orthopaedic Hospital of Shandong Province, Weihai, Shandong, China; ^3^ Department of Medicine, Chifeng University, Chifeng, China; ^4^ Department of Library, Children's Hospital Affiliated to Shandong University, Jinan, Shandong, China; ^5^ Department of Library, Jinan Children's Hospital, Shandong, Jinan, Shandong, China; ^6^ Department of Intensive Medicine, Wuxi People’s Hospital Affiliated to Nanjing Medical University, Wuxi, Jiangsu, China

**Keywords:** inflammation, immunity, cytokines, chemokines, pulmonary hypertension, immunosuppressive therapy

## Abstract

Pulmonary hypertension (PH) is a malignant disease with progressive increase of pulmonary vascular pressure, which eventually leads to right heart failure. More and more evidences show that immune cells and inflammation play an important role in the occurrence and development of PH. In the context of pulmonary vascular diseases, immune cells migrate into the walls of the pulmonary vascular system. This leads to an increase in the levels of cytokines and chemokines in both the bloodstream and the surrounding tissues of the pulmonary vessels. As a result, new approaches such as immunotherapy and anti-inflammatory treatments are being considered as potential strategies to halt or potentially reverse the progression of PH. We reviewed the potential mechanisms of immune cells, cytokines and chemokines in PH development. The potential relationship of vascular cells or bone morphogenetic protein receptor 2 (BMPR2) in immune regulation was also expounded. The clinical application and future prospect of immunotherapy were further discussed.

## 1 Introduction

Pulmonary hypertension (PH) is a condition characterized by changes in the structure and function of the pulmonary vasculature, resulting in an increase in pulmonary vascular resistance and arterial pressure (1, 2). If left untreated, PH can progress to a severe form of right heart failure or even death. The underlying pathophysiology of PH is complex and not yet fully understood. However, it is widely accepted that the development and progression of PH are closely associated with vascular remodeling (3). In physiological state, the contraction and relaxation of blood vessels maintain a balanced state (4, 5). When immune inflammatory reaction occurs, various inflammatory factors and oxygen free radicals are produced to damage endothelium, leading to its dysfunction (6). The balance between vasodilating substances such as NO and vasoconstricting substances such as endothelin (ET) is broken, and the contractility of pulmonary blood vessels is abnormally increased (7–9). It is reported that the thickness of intima, media and adventitia and the average pulmonary artery pressure are correlated with the average perivascular inflammation score, which supports the role of perivascular inflammation in pulmonary vascular remodeling (10). In addition, the study also showed that the inflammatory pathology is more advanced when the bone morphogenetic protein type 2 receptor (BMPR2) is mutated. In the experimental PH, the fact that inflammation precedes vascular remodeling indicates that immune changes are the cause of vascular diseases rather than the result (11).

**Figure 1:**
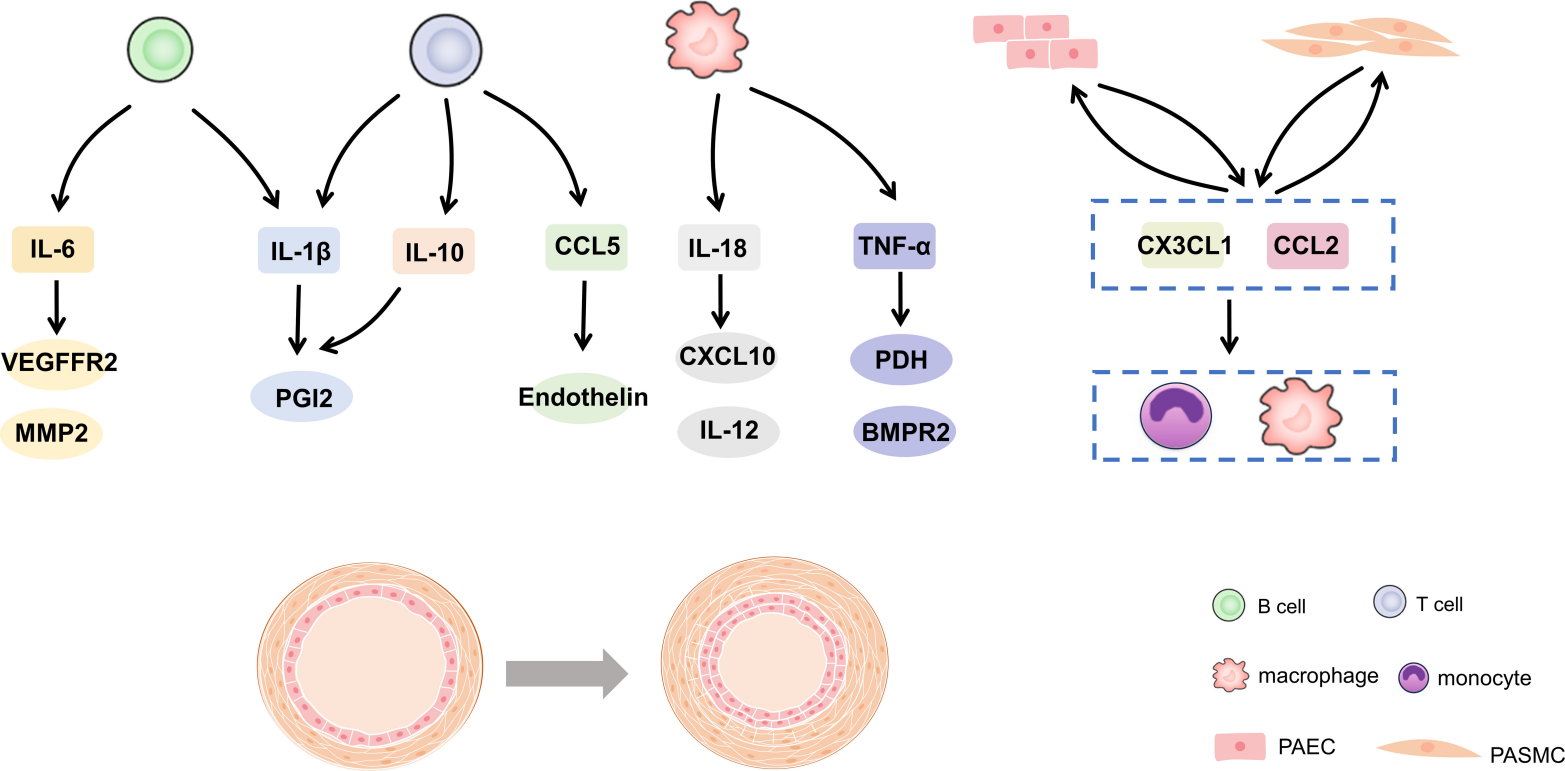
Cytokines and chemokines in pulmonary hypertension. IL, interleukin; CCL, C-C motif chemokine ligand; TNF, tumor necrosis factor-alpha; PGI2, prostacyclin; VEGFR2, vascular endothelial growth factor receptor 2; MMP, matrix metalloprotein; CXCL, C-X-C motif chemokine ligand; PDH, pyruvate dehydrogenase; BMPR2, bone morphogenetic protein type 2 receptor.

Further analysis of immune function in patients with PH proved that immune response exists in the process of PH occurrence and development (12). This can explain the accumulation of inflammatory cells around vessels and the excess of cytokines and chemokines (13, 14). In reality, maintaining a delicate balance between immunity and tolerance is crucial. Any disruption to this balance can result in chronic inflammation or even autoimmune disorders (15, 16). This review summarized the evidence and potential mechanism of immune cells and inflammation in PH in recent years. In addition, the potential relationship between vascular cells or BMPR2 in immune regulation was expounded. It is suggested that immunity and inflammation may be the key factors and promising therapeutic targets for PH development in **Figure 1**.

## 2 Immune cells and PH

Pulmonary vascular remodeling in pulmonary arterial hypertension (PAH) patients and PH animal models is often accompanied by different degrees of perivascular inflammatory infiltration, including T cells, B cells, macrophages, dendritic cells (DC), mast cells and neutrophils. This suggested that these immune cells may play an important role in the process of pulmonary vascular remodeling.

### 2.1 T cell

T cells are an important part of adaptive immune response and play an important role in the pathogenesis of PH, mainly helper T cells (Th cells) and regulatory T cells (Tregs) (17). Different types of T cells have specific functions and reactions in the inflammatory cascade reaction. Th cells produce pro-inflammatory response, while Tregs play a balanced response to achieve self-tolerance and prevent autoimmune (18). Studies have confirmed that the balance and homeostasis of T cells and their cytokines can prevent the loss of self-tolerance, and then affect the development of inflammation and PAH.

Based on the pro-inflammatory substances they release, Th cells can be categorized into different subsets, including Th1, Th2, and Th17 cells. Th1 and Th17 cells are capable of promoting inflammation through the production of various cytokines such as interleukin-6 (IL-6), IL-2, IL-21, interferon-gamma (IFN-γ), and tumor necrosis factor-alpha (TNF-α) (19, 20). The levels of peripheral Th17 cells, cytokines, and mRNA in patients with connective tissue diseases-associated PAH (CTD-PAH) were elevated (21). More importantly, the Th17/Tregs ratio was significantly related to the severity and prognosis of CTD-PAH (21). Moreover, the expression of T cell activation markers CD86 and CD40 was higher in idiopathic PAH (IPAH) patients following pretreatment with dexamethasone (22). Both with and without dexamethasone, PAH monocyte-derived DCs induced a higher activation and proliferation of CD4+ T cells, associated with a reduced expression of IL-4 (Th2 cells response) and a higher expression of IL-17 (Th17 cells response) (22). Animal studies have demonstrated that depleting CD4 cells or using SR1001, a Th17 cells development inhibitor, prevented the elevation of pressure and remodeling responses to chronic hypoxia (23). Furthermore, in a mice model of hypoxia-induced PH (HPH), the use of the receptor antibody MR16-1 to block IL-6 showed promising results. This treatment not only improved the condition of PH but also hindered the accumulation of macrophages and Th17 cells in the lungs (24).

And Th2 cells produce IL-4 and IL-13, which are described as antagonists of autoimmune pathological development. Researchers identified that Th2 CD4 T cells were necessary for Schistosoma-induced PH, given that deletion of CD4 T cells or inhibiting their Th2 cells function protected against type 2 inflammation and PH following Schistosoma exposure (25). They also observed that adoptive transfer of Schistosoma-sensitized CD4 Th2 cells was sufficient to drive type 2 inflammation and PH (25). Besides, it found that chemoattractant receptor homologous molecule expressed on Th2 cell (CRTH2) expression was up-regulated in circulating CD3CD4 T cells in patients with IPAH and in rodent PAH models (26). CRTH2 deficiency suppressed Th2 cells activation, including IL-4 and IL-13 secretion. CRTH2 disruption dramatically ameliorated pulmonary artery remodeling and PH in different PAH mice models (26).

Tregs play an important role in regulating the inflammatory response of Th cells to their own and foreign antigens. Tregs not only controls other T cells, but also regulates monocytes, macrophages, DCs, natural killer cells and B cells. In thymus-free rats with T cell immunodeficiency, pulmonary arterioles were blocked by proliferating endothelial cells. Blood vessels were surrounded by mast cells, B cells and macrophages, similar to human PAH (27). In addition, Tregs can regulate the proliferation of human pulmonary artery smooth muscle cells (HPASMCs). Tregs treatment significantly reduced the increase of right ventricular systolic pressure (RVSP) and Fulton index induced by hypoxia, decreased the expression of pro-inflammatory cytokines, and increased the level of IL-10 *in vivo*. This was attributed to the fact that Tregs treatment decreased the proliferation of HPASMCs and the expression of cyclin D1, cyclin-dependent kinase 4, p-Akt and p-ERK, and increased the expression of p27 *in vitro (* 28). Furthermore, Tregs can inhibit the accumulation of collagen by inhibiting the secretion of transforming growth factor (TGF)-β1 and fibroblast growth factor 9 (29). Tregs also down-regulated cardiac fibroblasts by secreting IL-10, which helped to control the development of right ventricular hypertrophy (RVH) in PAH (30).

### 2.2 Macrophage

Macrophages play a crucial role as a key component of the innate immune system, and the produced antigen is presented to T cells to differentiate and activate adaptive immune system. Studies have shown that pulmonary inflammation mediated by peripulmonary macrophages is the key factor of pulmonary vascular remodeling. In PAH rat model and patients with left heart disease-related PH (LHD-PH), compared with the control group, macrophages increased, accompanied by an increase in lung IL-6 (31). At the same time, in the mice model of chronic thromboembolic PH (CTEPH), F4/80 positive monocytes/macrophages obviously accumulated in high-flow arteries (32). In human and animal models with PH, it has been reported that the level of monocyte recruitment chemokines in the lung is increased, and the number of peripheral blood monocytes is increased. Upon migration to the pulmonary vascular system, monocytes had the potential to differentiate into perivascular macrophages. This process was facilitated by the activation of chemokines chemokine ligand 2 (CCL2) and C-X3-C motif chemokine ligand 1 (CX3CL1) (33). Moreover, the recruitment and activation of macrophages around pulmonary vessels have an impact on the function of pulmonary vascular cells. SU5416 induced PH in athymic rats, and macrophages gathered around pulmonary arterioles and synthesize excessive leukotriene B4 (LTB4). LTB4 can damage endothelial cells (ECs) of nearby blood vessels, cause apoptosis, and cause abnormal proliferation of SMCs. Blocking macrophage-derived LTB4 biosynthesis or signal transduction can reverse the experimental PH, while depletion of CD68+ macrophages can prevent the occurrence of PH in thymus-free rats treated by sugen (34). Similarly, the development of HPH was related to the significant increase of levels of CX3CR1, CC chemokine receptor 2 (CCR2) and their respective ligands CX3CR1 and CCL2. CX3CR1 deficiency can prevent HPH by regulating monocyte recruitment, macrophage polarization and PASMCs proliferation (35).

### 2.3 B Cell, DC and neutrophil

B cells have the ability to differentiate into plasma cells, which are responsible for producing autoantibodies. In addition, B cells play a crucial role in immune responses by collaborating with antigen-presenting DCs and lymphoid organs. They achieved this through antigen presentation, the production of various cytokines, and the facilitation of T effector cell differentiation (31, 36). On the one hand, a functional role for B cells in PH was demonstrated in that either blocking B cells by an anti-CD20 antibody or B cells deficiency in JH-KO rats attenuated right ventricular systolic pressure and vascular remodeling in experimental PH (32). On the other hand, B cell depletion therapy was a potentially effective and safe adjuvant treatment for systemic sclerosis-PAH and systemic lupus erythematosus-PAH (37, 38).

In monocrotaline (MCT)-induced rats and hypoxic mice, the accumulation of pulmonary neutrophils increased (39, 40). Although neutrophils have received little attention in the pathogenesis of PAH, it has been found that neutrophil elastase can affect the pathogenesis. Neutrophil elastase isolated from PAH patients, PASMCs and PH rat models was enhanced (41–44). In MCT-induced and Sugen/hypoxic rat models, after treatment with elafin, an inhibitor of neutrophil elastase, the changes of pulmonary intima subsided and the lumen size increased (39).

The study revealed that the number of circulating myeloid DCs in IPAH patients was lower than that in the control group, and the immune deficiency of monocyte-derived DCs was obvious (45). However, the circulating plasma DCs (pDCs) value of IPAH patients did not change, and the pDCs value in lung tissue increased (46). pDCs secretes inflammatory factors and chemokines to promote the activation of immune cells. At the same time, these inflammatory factors and chemokines attracted monocytes to the lungs of patients with IPAH and CTD-PAH, and produced monocyte-derived DCs, which further aggravated inflammation (47).

## 3 Cytokine and PH

The increase of perivascular immune cell accumulation and intravascular infiltration, accompanied by the abnormal increase of some cytokines, leads to the increase of vascular cell inflammation and the formation of dysfunction. They played crucial roles as regulatory factors in PAH and are closely associated with the disease’s severity (48). At the same time, the circulating level of cytokines was an important marker for the diagnosis and treatment of PAH (49). Targeting specific cytokine responses and pathways is considered a promising therapeutic strategy.

Some cytokines and chemokines were related to the poor clinical outcome of PAH patients, and may be used as biomarkers of disease progression. Some, such as IL-1β and TNF-α, were related to the accumulation of extracellular matrix proteins (such as fibronectin), which were found in PAH lesions, while others, such as IL-6, are related to the proliferation of SMCs.

### 3.1 IL-1β

A study has provided evidence that the levels of IL-1β are significantly elevated in patients with PH. This increase in IL-1β had been found to be associated with a poorer prognosis for individuals with PH (50). The mice model of PH showed the same conclusion, and it has been proved that starting IL-1β receptor antagonist can reduce PH and RVH (51). Furthermore, there existed a correlation between IL-1β and prostacyclin (PGI2) (17, 52). IL-1β increased PGI2 production in a dose-dependent manner (52). Cyclooxygenase-2 (COX-2) was the key enzyme for PGI2 synthesis (52). After treating PASMCs with IL-1β, the level of COX-2 mRNA increased, which induced the expression of PGI2 (52, 53). However, IL-1β has been found to have a negative impact on adenosine cyclase, leading to a decrease in cyclic adenosine monophosphate (cAMP) production. This, in turn, weakened the role of PGI2 (54). Furthermore, IL-1β activated other cytokines and chemokines to play a role. The cleavage of IL-18 by IL-1β invertase resulted in the production of bioactive IL-18. This increase in bioactive IL-18 has been observed in patients with pulmonary vascular diseases. Additionally, the levels of the downstream chemokine CXCL10 were also elevated in these patients (55). The increased expression of IL-18 and CXCL10 may make the inflammatory environment permanent and eventually lead to the vascular occlusion characteristics of PAH (55).

### 3.2 IL-6

IL-6 is a cytokine secreted by lymphocytes, which has extensive pro-inflammatory properties and participates in the occurrence and development of PH. IL-6 has been found to have prognostic value in patients with PH. The serum level of IL-6 has been shown to be an independent predictor of survival in PH patients and can be used to predict the outcome of these patients (56). Animal model revealed that the RVH of rats injected with recombinant human IL-6 increased, and PH appeared under normal oxygen conditions (57). Similarly, lung-specific IL-6 overexpression mice spontaneously produced PH under chronic hypoxia, and showed muscular distal arterioles and proliferative lesions in vascular cells. The IL-6 receptor knockout rats were not affected by HPH, and the progressive accumulation of PASMCs was reduced (58).

IL-6 may promoted PH in the following ways: (1) IL-6 induced excessive proliferation of pulmonary artery ECs (PAECs) and PASMCs in the distal pulmonary vascular wall by up-regulating vascular endothelial growth factor receptor 2 (VEGFR2) and matrix metalloprotein -2 (20). (2) IL-6 affected many signal pathways, such as BMPR2/Smad, MAPK/P38 and MAPK/JNK (17). (3) IL-6 played a crucial role in regulating the balance between Th17 cells and Treg cells. It can stimulate the immune response of Th17 cells while inhibiting the suppressive function of Treg cells. This imbalance in the immune response, with an increased presence of Th17 cells and reduced Treg cell activity, has been associated with an increased risk of PAH (59).

### 3.3 IL-10, IL-18 and TNF-α

Abnormal levels of cytokines, including IL-10, IL-18, and TNF-a, have been observed in both human and animal models of PH.

IL-10 is released by T cells, which is negatively correlated with PGI2 therapy. And IL-10 was decreased in patients after cardiopulmonary bypass (60). At the same time, it has been proved that the decrease of IL-10 level is a risk factor for chronic obstructive pulmonary disease (COPD)-PH (61). However, exogenous IL-10 significantly reduced macrophage infiltration and vascular cell proliferation in the remodeled pulmonary artery. It also significantly reduced the lung levels of TGF-β1 and IL-6 (62). Therefore, the effect of IL-10 on the development of PH needs to be studied.

Under the influence of caspase-1, the inactive precursor form of IL-18 is converted into its active form, IL-18, which possesses biological activity (55). IL-18 acted on the cytokine-chemokine cascade reaction of type I immune response, affecting the expression of IL-12, IFN-γ and CXCL10. This promoted the recruitment of more lymphocytes to the vascular wall, which in turn exacerbated the pathological progression of PAH (55). Besides, IL-18 affected SMCs in an autocrine or paracrine way. IL-18 could thicken the smooth muscle layer by up-regulating the chemotaxis of metalloproteins and cells that constitute the vascular skeleton (63).

TNF-α is an important proinflammatory factor, which is significantly increased in the medial layer of pulmonary artery in COPD-PH patients (64). It is reported that transgenic mice that overexpress TNF-α will have severe PH value and right ventricular hypertrophy (65). Similarly, injection of TNF-a into the rat model led to increased vascular activity and remodeling (66). TNF-α may drove the proliferation of PAECs and PASMCs in HPH by inhibiting pyruvate dehydrogenase (PDH) activity, inhibiting BMPR2 and changing NOTCH signal transduction (63).

## 4 Chemokine and PH

### 4.1 CCL2

CCL2 is secreted by vascular ECs and SMCs, and its role is mediated by CCR2. CCL2 is an effective medium for the activation of monocytes and macrophages, which induces the secretion of cytokines and the expression of adhesion molecules (17, 67). It is reported that CCL2 induces the proliferation and migration of PAECs and SMCs in patients with PH, which leads to vascular remodeling and blood pressure increase (68). This may be because the increase of CCL2 produced by pulmonary vascular cells contributes to the increase of chemotactic activity of monocytes/macrophages. So that adventitial fibroblasts are activated (69, 70), the migration and proliferation of PASMCs increased, and pathological vascular remodeling was induced. In the presence of pulmonary ECs, the mobility of monocytes decreased significantly, while the blocking antibody of CCL2 increased significantly. The migration and proliferation of PASMCs in patients with PAH increased in response to CCL2 stimulation (71).

### 4.2 CCL5

CCL5 is expressed and secreted by T cells (17). Studies have confirmed that CCL5 can promote the proliferation of ECs. ET-converting enzyme-1 and ET promoted mitosis and vasoconstriction, and accelerated the pathological process of PH (72). In fact, compared with the control group, the expression of CCL5 mRNA in lung samples of PAH patients was increased, which may be derived from ECs (73). Moreover, the crosstalk between CCR5 and CCR2 mediated the synergy between macrophages and PASMCs, thus promoting inflammatory cell infiltration and PASMCs migration and proliferation during the development of PAH (74).

### 4.3 CX3CL1

CX3CL1 exists as a cell adhesion molecule on ECs or as a chemotactic protein, and its function is mediated by CX3CR1 (67). Under the pathological condition of PH, CX3CL1 led to the adhesion of macrophages expressing CX3CR1 to PAECs, which led to PAECs dysfunction and trigger perivascular inflammatory reaction (75). In addition, CX3CL1 promoted the proliferation of pericytes and PASMCs, which further aggravated the remodeling of pulmonary small vessels, leading to further aggravation of PH (76). CX3CR1 deficiency protected against HPH by modulating monocyte recruitment, macrophage polarization, and PAMCs proliferation (35).

## 5 Vascular cells and BMPR2

### 5.1 Vascular cells

ECs play a key role in maintaining vascular homeostasis under various stimuli, and regulate inflammation through mediators such as NO, ET, cell adhesion molecules, cytokines and chemokines. Under pathological conditions such as inflammation and hypoxia, PAECs can reduce the production of vasodilators (such as NO) and vascular growth factors, which is beneficial to the vasoconstriction of the distal pulmonary artery (77). Circulating ECs may be involved in the process of vascular injury, tumorigenesis or interaction with immune cells. In addition, endothelial progenitor cells were bone marrow-derived cells involved in homeostasis, and they were also physiological and pathological angiogenesis cells. The increase of proinflammatory cytokines was also beneficial to the activation of platelet adhesion and coagulation cascade reaction, which led to further occlusion of arterioles (78).

It has been suggested that inflammation can recruit SMCs population and enhance their contribution to pulmonary vascular remodeling. Because excessive proliferation of SMCs was observed in locally occluded blood vessels. Some studies also showed that continuous hypoxia can induce the recruitment of mesenchymal progenitor cells around blood vessels. The recruitment of these cells was very important for the occurrence of PAH (79) [142,143]. The changes of inflammatory vascular system may be caused by these migrating cells and resident SMCs, which restore the ability needed for vascular remodeling.

### 5.2 BMPR2 and immune

On the one hand, BMPR2 gene mutation has been identified as the main genetic cause of PAH. On the other hand, BMPR2 plays an important role in maintaining the immune system (80, 81). The disruption of BMPR2 signaling pathway causes an increased degree of inflammation and decreases the ability of the immune system to resolve it. Inhibition of BMPR2 gene expression led to unregulated proliferation and survival of endothelial cells through disordered TGF-β signaling, thus promoting vascular remodeling (82). In order to cope with the decrease of BMPR2 function in ECs, it is assumed that the integrity of ECs dysfunction may be damaged, which may lead to apoptosis, the release of TGF-β and the development of anti-apoptotic clones (83). On the contrary, SMCs proliferate due to TGF-β signaling, and undergo excessive growth reaction, leading to vascular remodeling (84).

Loss of BMPR2 expression enhanced ECs inflammatory response through various mechanisms mediated by ROS, nicotinamide adenine dinucleotide phosphate oxidase and NF-κB activity (85, 86). In innate immune response, BMPR2 deficiency increased the recruitment of macrophages to the perivascular area of pulmonary vascular. Mutated BMPR2 can also increase the production of granulocyte macrophage colony-stimulating factor, thus activating macrophages (87, 88). Activated macrophages secreted proinflammatory cytokines, such as IL-6 and IL-8 (86). In addition, the imbalance of BMPR2 in adaptive cellular immunity will destroy the development of T cells and lead to the increase of ECs apoptosis. The decrease of BMPR2 expression was also related to CD4 T cells depletion. Lack of T cells population led to inflammation around the artery, which was dominated by macrophage and B cells activity, thus aggravating PAH (11, 46).

## 6 Immunosuppression therapy in PH

At present, it is generally believed that there is a connection between inflammation and PH, but there are still several problems. Firstly, it is not clear whether inflammation is enough to promote the development of PH. Secondly, what are the causes of immune and inflammatory changes in PH lung tissues? Finally, does this have pathological or clinical significance? It is necessary to better understand the mechanism of immune cells, cytokines and chemokines in PH affecting abnormal angiogenesis and pulmonary artery remodeling. Although it is necessary to understand the immune/inflammatory components of PH more clearly, recent studies have shown promising results in animal models regarding the efficacy of anti-inflammatory therapies in reducing or even reversing the effects of PH. Currently, therapeutic drugs targeting the three pathological pathways of PH primarily focus on vasodilation. However, it is worth noting that these drugs also exhibit immunomodulatory properties (89). At present, there is no approved treatment specifically targeting the inflammatory process associated with pulmonary vascular diseases.

Tacrolimus is a calcineurin inhibitor, which can regulate immunity and has anti-inflammatory activity. Low-dose tacrolimus has been proved to reverse the disease progression of PAH rats induced by mc and hypoxia, restore the normal function of pulmonary artery endothelial cells, and activate other functions of BMPR2 receptor signal transduction by removing FKBP12 from BMPR 1 type co-receptor (90).Based on B cell depletion therapy, patients who received rituximab (an anti-CD20 monoclonal antibody) showed a decrease in rheumatoid factor, IL-12 and IL-17 (37). Hydroxymethylglutaryl coenzyme A (statins) have been proved to have anti-inflammatory and immunomodulatory functions (91, 92). Patients with IPAH, HPAH and CTD-PAH were treated with simvastatin, which inhibited lymphocyte function and improved right ventricular remodeling (93, 94). In addition, compared with the control group, the level of circulating inflammatory markers in patients with chronic embolic PH and PAH increased (95). After treatment with corticosteroids or immunosuppressants, the clinical symptoms of some patients improved (96). The above evidence reveals that suppressing immunity and inflammation can be used as a potential strategy for PH treatment. However, immunosuppressants are a kind of drugs that can effectively inhibit immune cell function, reduce immune response and inhibit inflammation. So far, no immunosuppressants have been approved for the treatment of multiple PH. The therapeutic effect of most immunomodulators on PH is still in the clinical trial stage.

## 7 Conclusion

It is an obvious fact that immune cells and inflammation play an important role in the pathophysiology of PH. There is increasing evidence that PH is not only caused by dynamic vasoconstriction. Inflammatory cells and their chemokines and cytokines affect pulmonary vascular system. However, many molecular and cellular mechanisms remain unsolved. It is significant for immunotherapy and anti-inflammation to better understand how inflammation and immunity participate in the development of polycyclic aromatic hydrocarbons.

## Author contributions

HZ: Conceptualization, Writing – original draft. JS: Investigation, Writing – original draft. XL: Investigation, Visualization, Writing – review & editing. ZX: Visualization, Writing – review & editing. QW: Writing – review & editing, Formal Analysis. JF: Writing – review & editing, Methodology. YM: Project administration, Writing – review & editing. DW: Conceptualization, Project administration, Writing – review & editing. XW: Conceptualization, Project administration, Writing – review & editing.
